# Implementing physical activity calorie equivalent (PACE) food labelling: Views of a nationally representative sample of adults in the United Kingdom

**DOI:** 10.1371/journal.pone.0290509

**Published:** 2023-09-14

**Authors:** Amanda J. Daley, Victoria E. Kettle, Andrea K. Roalfe

**Affiliations:** Centre for Lifestyle Medicine and Behaviour (CLiMB), The School of Sport, Exercise and Health Sciences, Loughborough University, Loughborough, United Kingdom; AIIMS Jodhpur: All India Institute of Medical Sciences - Jodhpur, INDIA

## Abstract

**Background:**

Nutritional labels aim to support people to make informed healthy food choices, but many people do not understand the meaning of calories on food labels. Another approach is to provide calorie information with an interpretation of what the calorie content of food means for energy expenditure, known as physical activity calorie equivalent (PACE) labelling. PACE aims to illustrate how many minutes of physical activity are equivalent to the calories contained in food/drinks. This study investigated the views of the public about the possible implementation of PACE labelling.

**Methods:**

Data was obtained from a nationally representative sample of adults in the United Kingdom and collected by UK Ipsos KnowledgePanel. Panellists are recruited via a random probability unclustered address-based sampling method. 4,000 panellists were randomly invited to participate and asked to compare their views about traffic light and PACE labelling preferences and behaviour parameters.

**Results:**

Data were analysed descriptively and using logistic and multinomial regression analyses. 2,668/4,000 (67%) of those invited participated. More participants preferred traffic light (43%vs33%) than PACE labelling, but more reported PACE was easier to understand (41%vs27%) and more likely to catch their attention (49%vs31%). More participants thought PACE was more likely to help them avoid high calorie food than traffic light labelling (44%vs28%). Physically active (3–4 or 5+ days/week) respondents were more likely to report PACE would catch their attention than traffic light labelling, compared with less active participants (weighted adjusted relative risk ratio = 1.42 (1.00–2.00) and 1.45 (1.03–2.05 respectively)). Perceived overweight was the most predictive factor (weighted adjusted OR = 2.24 (1.19 to 4.20)) in whether PACE was considered useful in helping people decide what to eat/buy.

**Conclusion:**

The public identified value to their health in labelling food with PACE information. PACE labelling may be a useful approach to complement current approaches to food labelling.

## Introduction

Overweight is a serious public health concern in many countries [1]. There has been no long-term success in reducing obesity rates and there is recognition this is, in part, is due to the physical environments that surround the public, which can exert considerable influences on health behaviors [2]. Nutritional labels are one way to support people to make more informed, healthier food choices and in many countries, including in the United Kingdom, a traffic light labelling system is the national standard [3]; this approach judges the nutritional quality of food by using red, amber and green codes on food packaging, along with the percentages that relate to the amount of salt, sugar, fat and saturated fat in the food item (see https://www.food.gov.uk/safety-hygiene/check-the-label) [3]. The World Health Organisation considers nutrition labelling an essential part of its global strategy on diet, physical activity and health [4]. However, there is limited evidence from randomised controlled trials showing that nutrition information on food/drinks is changing the purchasing or consumption behaviours [5–7]. Furthermore, the evidence that does exist, has reported small effect sizes [8]. Many people do not understand the meaning of kilocalories (kcals or calories) or grams of fat displayed on food labels, and often underestimate the number of calories when labelling is not provided [9, 10]. Therefore, a key challenge to limiting energy consumption may be the significant underestimation by the public of the number of kcals in food/drinks, and its relationship to their energy needs. There may be more effective ways to present calorie information to increase their impact and reduce rates of overweight and obesity in the population.

### Physical activity calorie equivalent (PACE) labelling

**Fig 1:**
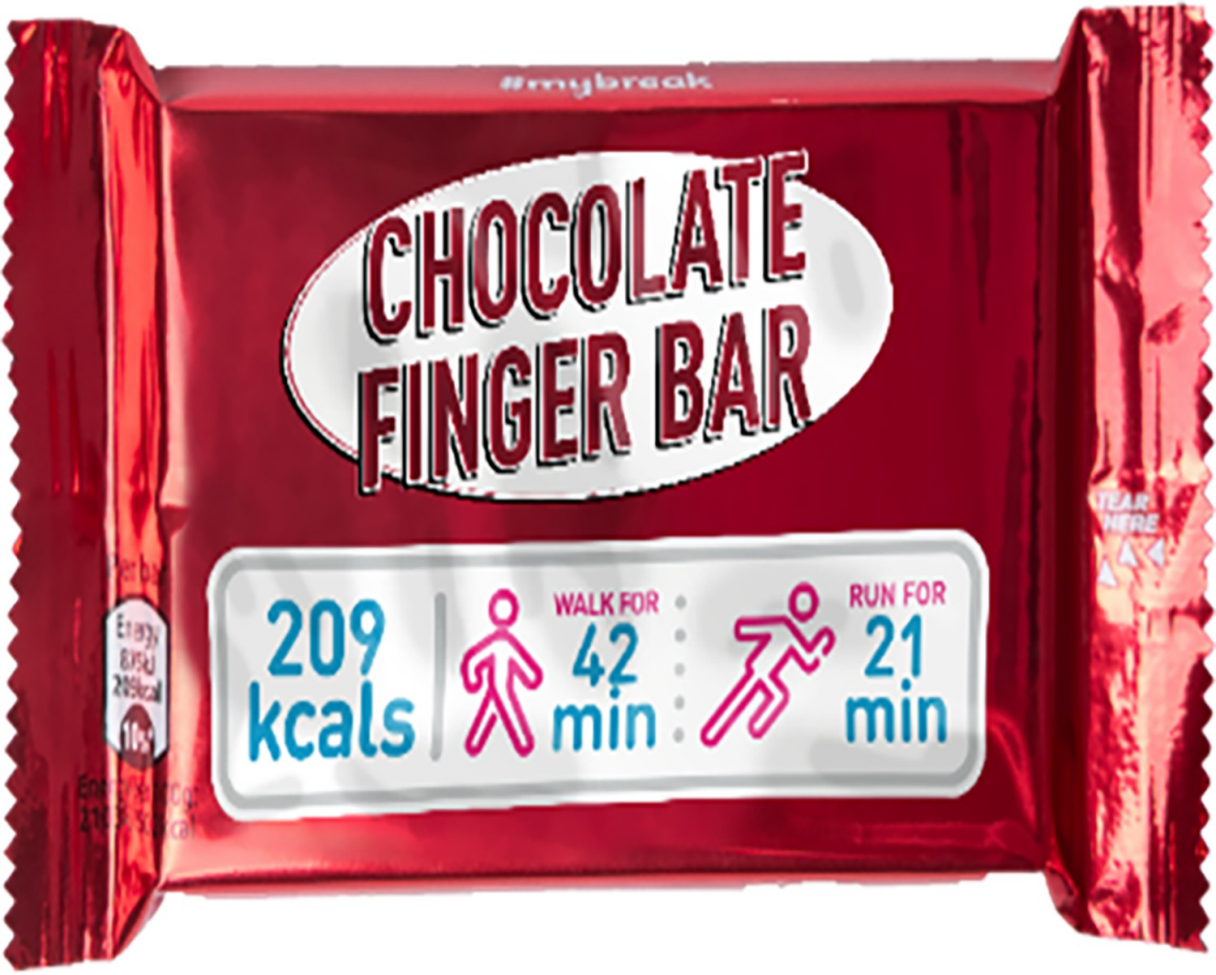
Example of PACE labelling (1).

**Fig 2:**
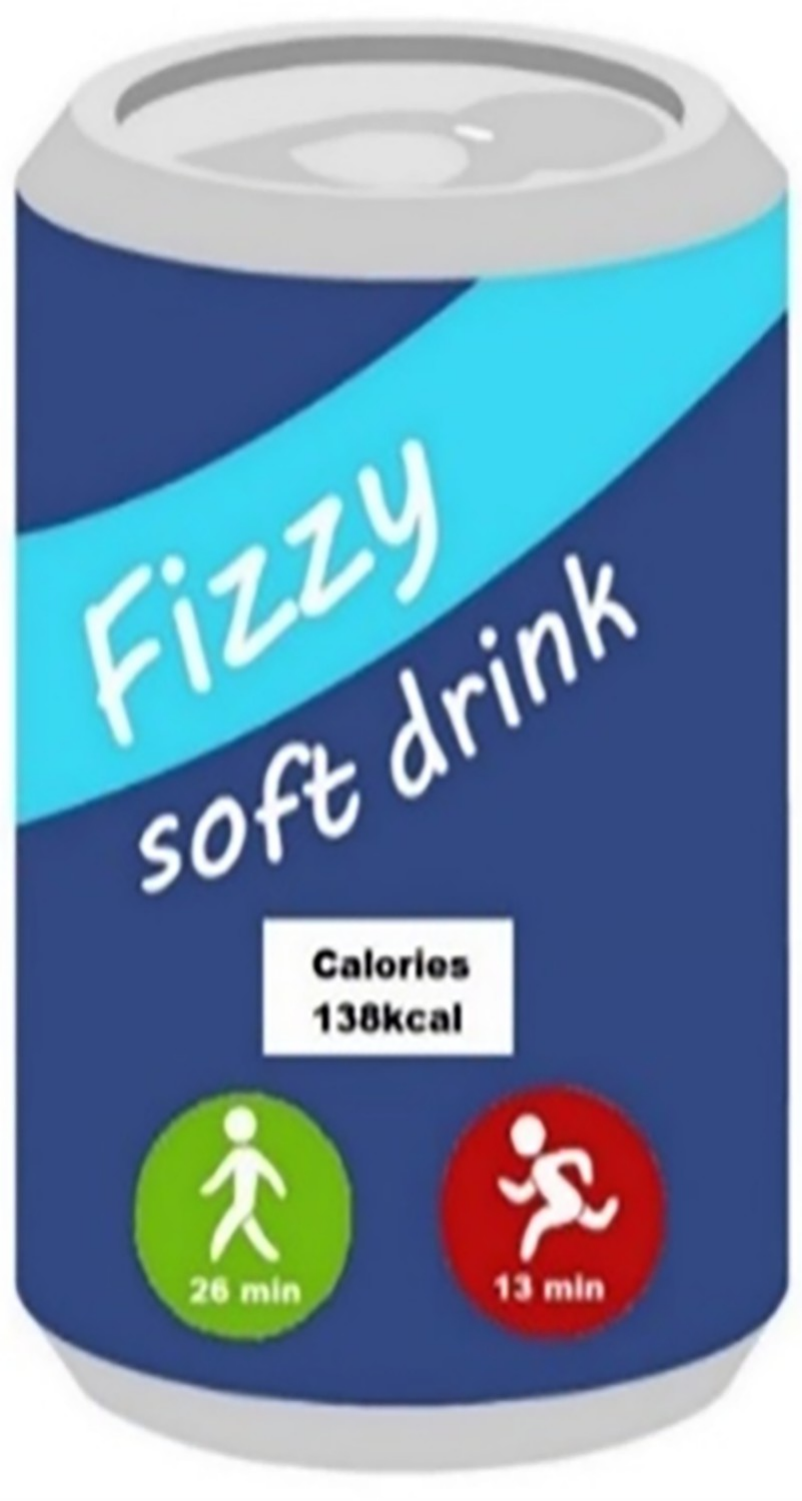
Example of PACE labelling (2).

Another approach to nutrition labelling, is to provide calorie information with an interpretation of what the calorie content of the food/drink means in terms of energy expenditure. This approach is referred to as physical activity calorie equivalent (or expenditure) labelling (PACE), which aims to illustrate how many minutes (or miles/kilometres) of physical activity are equivalent to the calories contained in food/drinks [11]. For example, “the calories in this chocolate cake requires 100 minutes of walking to burn off” (Figs 1 & 2). PACE labelling could be useful in helping the public understand what energy content means by providing a context for the number of calories in food/drinks, enabling them to decide whether the calories are worth the amount of activity required to expend the calories. It is suggested that PACE labelling could more readily catch consumers’ attention and it allows for rapid comprehension of calorie information when making food purchase and consumption decisions. PACE labelling has the potential to serve as a frequent reminder or nudge to the public about the importance of participating in physical activity to maintain good energy balance [12]. Increasingly, governments are mandating that the calorie content of foods/drinks should be displayed in out of home settings, and there could be an opportunity to include PACE labelling within this implementation [13, 14].

### Current study

There is some evidence that PACE labelling may be effective in reducing the average number of calories selected for consumption by the public, but there is a lack of evidence about the views of the public about possible implementation [11]. It is important to understand the views of the public about the implementation of PACE labelling to maximise any potential impact and to guide decisions concerning where to allocate resources to facilitate any such impact. Furthermore, it is not known whether the public have location preferences for different types of labelling (e.g., vending machines, supermarkets & fast food outlets), and which types of food/drinks they would like to see PACE labelling displayed, to inform their food choices. It is also not clear what concerns the public may have about the use of PACE labelling. This study aimed to address these questions to help guide future health policy about the role of PACE labelling.

## Methodology

### Participant recruitment and sample

This cross-sectional study obtained data from a nationally representative sample of adults from across the United Kingdom recruited via the UK Ipsos Knowledge Panel (https://www.ipsos.com/en-uk/uk-knowledgepanel) [15]. Panellists are recruited via a random probability unclustered address-based sampling method where every household in the UK has a known chance of being selected to join the panel. Letters are sent to selected addresses in the UK inviting them to become panel members. Up to two household members are allowed to sign up to the panel. Members of the public who do not have access to the internet can register to the Knowledge Panel either by post or by telephone, and are given a tablet, an email address, and basic internet access which allows them to complete surveys online. These approaches not only improve population coverage, but also provide a more effective means for recruiting hard-to-reach individuals, such as those without landline telephones or computer/internet access. At the time of joining the Knowledge Panel, panellists completed a general online administered informed consent process and were reminded of their rights to privacy and to withdraw at any time. As the Knowledge Panel is a random probability survey, panel invited samples are stratified when conducting waves to account for any profile skews within the panel. At the time of this study the Knowledge Panel contained 14,016 available panellists and of these 4,000 were randomly selected and invited to take part in this study. The sample was stratified by region and education. The sample drawn was then reviewed against population statistics on ethnicity, index of multiple deprivation (IMD) [16], urbanity of home postcode (rural vs urban), age and gender.

### Ethics

This study was conducted according to the guidelines laid down in the Declaration of Helsinki and all procedures involving research study participants were approved by the Loughborough University Ethical Committee for Human Participants (reference number: 5523). Written informed consent was obtained from all participants.

### Study survey

This study was conducted between 24–30 June 2021. At the time of joining the Knowledge Panel, panellists were asked to complete a core survey profile, which included questions about their age, gender, ethnicity, income, country of residence, highest level of education, annual household income and physical activity behaviour. Data regarding participants’ perception of their weight status (underweight, about the right weight & overweight) were also collected in this study. Questions related to participants self-reported height and weight were part of a parallel survey that participants completed, and these data was included here. Panellists received 50 Knowledge Panel points for completing this study (£0.50 towards a shopping voucher).

Participants were asked their views about traffic light food labelling as the comparison label type because this is a commonly used type of food labelling in several countries in supermarket settings, including the UK where this study took place. As such traffic light labelling provided participants with a context and a familiar label type, in which to compare their views about PACE labelling. Participants were also asked their preferences for which foods, drinks, and locations they thought PACE labelling would be most useful/helpful. Participants were asked to compare their views about traffic light and PACE labelling as regards to which they preferred, easier to understand, caught their attention the most, and more likely to help them avoid high calorie food/drinks. Participants were given the opportunity to provide free text comments about their preferred type of labelling and any additional views they wished to make. When there were more than 10 possible response options to a given question, these were presented in a random order to respondents. All data received by the University research team were de-identified.

### Analysis

To ensure the findings were representative of the UK population, the below weighting specification was applied to the data in line with the target sample profile. As only two members per UK household are allowed to register with the Knowledge Panel, we employed a design weight to correct for unequal probabilities of selection of household members. Calibration weights were applied using the latest population statistics relevant to the surveyed UK population. England and Wales, Scotland and Northern Ireland were each weighted separately while an additional weight was created for the UK to account for any over or under sampling within each of these countries. Two sets of calibration weights were applied. Calibration weighting was applied using region and an interlocked variable of gender by age (both use Office of National Statistics 2020 mid-year population estimates as the weighting target [17]. Demographic weights were then applied to correct for imbalances in the achieved sample and the data weighted on education, ethnicity, IMD (quintiles), and number of adults in the household. Estimates from the ONS 2020 mid-year population estimates and Annual Population Survey were used as the weighting target [17].

Descriptive statistics were generated for each question in the survey. All statistics are reported as unweighted frequencies and weighted percentages to maximize transparency, unless indicated otherwise as ‘weighted results. All respondents were included in the analysis, but percentages are reported for each survey item only among those who answered the item. Thus, missing responses are removed from the denominator. Physical activity status was calculated according to the number of times per week respondents participated in at least moderate intensity physical activity (defined as enough to raise their breathing rate, including sport, exercise, and brisk walking or cycling for recreation or to get to and from places, excluding housework or occupational physical activity).

For data analysis weighted logistic regression models were used to identify significant predictors of support in relation to the key questions. Independent variables included in the modelling were age (18–34, 35–44, 45–54, 55–64 & 65+ years), gender, ethnicity (white & ethnic minorities), IMD quintile, perception of weight category (underweight, about right & overweight), physical activity (days per week, 0, 1–2, 3–4, 5+). Two way and three-way interactions were also explored but were not included in the final models due to compilation errors caused by sparsity of data. Standardised coefficients were calculated to compare characteristics most likely to support each question of interest.

Comparison of traffic light and PACE labelling preference, ease of understanding, attention, avoidance of high calorie food were compared across age category, gender, perception of weight status and physical activity status. Multinomial modelling was used to identify which of these four characteristics had an independent effect on each outcome after allowing for all others. Unweighted analysis and analysis excluding ‘don’t know’ responses were carried out as sensitivity analysis (not reported but available for the first author). Cross tabulation of multiple responses was conducted to understand participants thoughts regarding the most popular location and food/drink combinations where they would like to see PACE labelling placed. The open text comments were thematically coded (S1 Material in S1 File).

## Results

### Participants and demographics

Of 4,000 individuals invited to participate, 2,668 surveys were completed (response rate of 67%). A total of 1,302 participants (48%) were male, 2,521 (89%) identified as White ethnicity and 672 (44%) were aged 18–44 years and 1,996 (56%) aged ≥ 44 years. Most participants (n = 1,625, 59%) perceived themselves to be overweight and 56% were classified as overweight/obese according to their BMI (calculated using self-reported height/weight.) See Table 1 for participant characteristics.

**Table 1:** Participant characteristics.

Socio-demographics characteristics	N	% weighted	% unweighted
**Gender**			
Male	1,302	48.2	48.8
Female	1,348	51.1	50.5
Other/prefer not to say	18	0.7	0.7
**Age**			
18–24	100	10.6	3.8
25–34	244	17.0	9.2
35–44	328	16.1	12.3
45–54	499	17.4	18.7
55–64	674	15.5	25.3
65–74	598	12.7	22.4
75+	225	10.7	8.4
**Ethnicity**			
White	2,521	89.2	95.4
Ethnic minority	119	10.8	4.5
**Education**			
Degree level or above	806	30.7	31.7
Below degree level	1,739	69.3	68.3
**Country**			
England	2,235	84.0	83.8
Scotland	239	8.4	9.0
Wales	121	4.8	4.5
Northern Ireland	73	2.8	2.7
**Deprivation (quintile) (IMD)**			
1 (most deprived)	397	19.2	14.9
2	470	20.0	17.6
3	558	20.0	20.9
4	591	20.3	22.2
5 (least deprived)	652	20.4	24.4
**Perception of body weight category**			
Underweight	101	4.8	3.8
About the right weight	925	35.8	34.9
Overweight	1625	59.4	61.3
**BMI category**			
Underweight	34	1.8	1.5
Healthy weight	942	42.1	41.0
Overweight/obese	1324	56.2	57.6
**Physical activity behaviour category**			
0 days	455	18.1	17.6
1–3 days	1086	42.6	41.9
4–5 days	555	21.9	21.4
6–7 days	494	17.4	19.1

% may not add up to 100% due to rounding. Data reported as unweighted frequencies and weighted percentages. Data related to valid percentages and does not include those who responded don’t know/prefer not to say.

### Views about traffic light food labelling and predictor variables

Thirty one percent (n = 876) of participants indicated they never/rarely looked at traffic light labels on food/drinks packaging to help them decide what to eat or drink, 31% (n = 784) sometimes looked and 38% (n = 1,003) often/always looked. In those who indicated they never/rarely looked at traffic light labels (n = 876), the most frequent reasons were not sure what I should be looking at (20%, n = 181), do not understand them/they are too complicated (23%, n = 189) and I never notice them (25%, n = 212). Most participants (71%, n = 1,860) indicated they thought traffic light labels were very easy/fairly easy to understand. Thirty nine percent (n = 1,052) reported traffic light labels never/rarely stopped them buying/eating foods and drinks high in calories, and 40% (n = 1,047) reported they sometimes did. Being female or being physically activity on five or more days/week were significantly associated with higher odds of reporting always or often using traffic light labelling to prevent the purchase of food/drinks high in calories (gender was the most predictive factor (weighted adjusted odds ratio (OR) 95% confidence interval (CI) = 1.85 (1.43 to 2.39))). See Table 2.

**Table 2:** Weighted logistic regression models for individual traffic light/PACE labelling questions.

Demographic Characteristic	Traffic light labels: to stop buying food/drinks high in calories	PACE labels: help decide what to buy/eat	PACE labels: not a good idea or a negative impact on relationship with food	PACE labels: need to burn off everything
	Weighted Adjusted Odds Ratio (95% CI)	Weighted Adjusted Odds Ratio (95% CI)	Weighted Adjusted Odds Ratio (95% CI)	Weighted Adjusted Odds Ratio (95% CI)
Age group				
18–34	1.00 (0.69 to 1.47)	**1.37 (1.01 to 1.87)**	**2.69 (1.67 to 4.35)**	**2.35 (1.71 to 3.22)**
35–44	0.70 (0.47 to 1.03)	**1.65 (1.20 to 2.26)**	**2.00 (1.22 to 3.26)**	**2.07 (1.50 to 2.84)**
45–54	0.87 (0.62 to 1.23)	**1.98 (1.50 to 2.61)**	1.23 (0.76 to 1.98)	**2.24 (1.70 to 2.95)**
55–64	0.90 (0.67 to 1.22)	**1.50 (1.18 to 1.91)**	1.16 (0.78 to 1.70)	**1.53 (1.19 to 1.96)**
65+ (reference)	1.00	1.00	1.00	1.00
Gender				
Male (reference)	1.00	1.00	1.00	1.00
Female	**1.85 (1.43 to 2.39)**	**1.48 (1.20 to 1.82)**	**1.50 (1.10 to 2.06)**	**1.64 (1.34 to 2.00)**
Ethnicity				
White (reference)	1.00	1.00	1.00	1.00
Ethnic minorities	0.98 (0.55 to 1.74)	**1.77 (1.05 to 2.98)**	0.52 (0.19 to 1.43)	**2.12 (1.27 to 3.55)**
IMD quintile				
1 most deprived	1.00	1.00	1.00	1.00
2	0.75 (0.49 to 1.13)	1.11 (0.77 to 2.00)	1.29 (0.74 to 2.23)	0.84 (0.59 to 1.21)
3	0.74 (0.49 to 1.13)	0.93 (0.66 to 1.33)	1.17 (0.69 to 1.98)	0.83 (0.59 to 1.18)
4	0.81 (0.55 to 1.20)	1.17 (0.83 to 1.64)	1.53 (0.90 to 2.60)	0.95 (0.68 to 1.33)
5 least deprived (reference)	0.89 (0.60 to 1.31)	**1.41 (1.01 to 1.98)**	0.84 (0.49 to 1.44)	1.00 (0.72 to 1.39)
Perception of weight				
Underweight (reference)	1.00	1.00	1.00	1.00
About right	1.21 (0.56 to 2.61)	1.52 (0.79 to 2.90)	1.66 (0.67 to 4.10)	1.04 (0.57 to 1.93)
overweight	1.50 (0.71 to 3.17)	**2.24 (1.19 to 4.20)**	2.24 (0.92 to 5.49)	1.59 (0.88 to 2.90)
Physical activity (days/week)				
0 (reference)	1.00	1.00	1.00	1.00
1–2	1.14 (0.78 to 1.69)	**1.57 (1.16 to 2.14)**	**1.64 (1.02 to 2.63)**	**1.47 (1.07 to 2.01)**
3–4	1.33 (0.90 to 1.97)	**1.51 (1.10 to 2.07)**	1.19 (0.74 to 1.92)	**1.68 (1.21 to 2.33)**
5+	**1.64 (1.12 to 2.40)**	**1.67 (1.23 to 2.27)**	1.39 (0.88 to 2.19)	**2.19 (1.60 to 2.99)**

Statistical significant effects (p<0.05) are in bold; CI: confidence interval.

### Views about PACE food labelling and predictor variables

Most respondents (83%, n = 2,208) reported that PACE labelling was very/fairly easy to understand and 59%(n = 1,549) thought that if PACE labels were placed on food/drinks, they would definitely/probably look at them to help them decide what to buy and eat. Of those who would not, or did not know if they would look at PACE labels (n = 1,119), the most common reasons given were as follows; did not think PACE labels were a good idea (29%, n = 310) and PACE labels would negatively impact their relationship with food (20%, n = 193). An additional 38% (n = 464) of participants indicated that they would not look at PACE labels for other reasons and these were thematically coded as follows (three most frequent reasons); PACE labelling lacked detailed nutritional information (e.g. saturated fat, sugar etc) (n = 102); participants felt they already understood which foods were considered healthy/unhealthy and their nutritional values (n = 74); PACE is meaningless/inaccurate because it is based on average person/energy expenditure rate (n = 46).

Of respondents who reported they would definitely/probably look at PACE labels to help them decide what to buy/eat (n = 1,549), 93% (n = 1,430) thought PACE labels would definitely/probably stop them or make them think twice about buying high food/drinks. Being under 65 years, female, non-white ethnicity, resident in the most affluent area (IMD quintile 5), perceived overweight or physically active at least 1 day/week increased the likelihood that PACE labels would be considered useful in helping people decide what to buy/eat (Table 2). Perceived overweight was the most predictive factor (weighted adjusted OR = 2.24 (1.19 to 4.20)). Being under 45 years, female and physically active at least one day/week increased the odds of not thinking PACE was a good idea, with being in the 18–34 age group the most important factor (weighted adjusted OR = 2.69 (1.67 to 4.35)). See Table 2. A total of 41% (n = 977) of participants thought PACE labels would definitely/probably encourage them to do more physical activity each day, with 47% (n = 1,337) reporting ‘not really’, and 10% (n = 311) responding it definitely would not encourage them to do more physical activity.

### Comparison of views about traffic light and PACE labelling and predictor variables

More participants preferred traffic light (43%, n = 1,178 vs. 33%, n = 859) than PACE labelling. Respondents aged 44–54 years were more likely to prefer PACE over traffic light labelling than the youngest group (weighted relative risk ratio (RRR) = 1.49 (1.02 to 2.16)). Compared to traffic light labelling, more participants reported PACE labelling was easier to understand (27%, n = 719 vs 41%, n = 1,063). None of the four characteristics of interest were associated with this question. More participants thought that PACE was more likely to catch their attention than traffic light labelling, (49%, n = 1,274 vs 31%, n = 847). Respondents who were physically active at least 3–4 times/week and ≥5 times/week were 40% more likely to report PACE labelling would catch their attention than traffic light labelling compared with participants who were active 0–2 times/week (weighted adjusted RRR = 1.42 (1.00 to 2.00) and 1.45 (1.03 to 2.05 respectively)). More participants thought PACE was more likely to help them avoid high calorie food/drinks than traffic light labelling (44%, n = 1,124 vs 28%, n = 780). PACE labelling was chosen less often by those aged 65 years or more compared to the youngest group (weighted adjusted RRR = 0.60 (0.42 to 0.87)). See S1 Table in S1 File and Table 3.

**Table 3:** Comparison of preferences for different types of food labels.

N (%)	Traffic light	PACE	About the same	Neither	Don’t know
Which do you prefer?	1178 (43.2)	859 (32.8)	421 (16.3)	183 (6.6)	27 (1.2)
Which is easier for you to understand?	719 (26.8)	1063 (40.9)	774 (28.4)	89 (2.9)	23 (1.0)
Which is more likely to catch your attention?	847 (30.8)	1274 (49.2)	368 (14.4)	159 (4.7)	20 (0.9)
Which is more likely to help you avoid high calorie food/drinks?	780 (28.3)	1124 (44.4)	408 (15.3)	331 (10.9)	25 (1.1)

Data are reported as unweighted frequencies and weighted percentages

### Preferred locations for PACE and types of food/drinks for PACE labelling

Respondents felt that the most useful/helpful locations to see PACE labels on food/drinks would be instore/online supermarkets (67%), fast food outlets (68%), takeaway/online menus (55%), vending machines (39%) and menus in restaurants (35%). Participants reported that it would be most useful/helpful to see PACE labelling placed on were chocolate bars (60%), cakes/pastries (57%), ready meals (54%), sweets (46%), crisps (41%) and biscuits (41%). For drinks, participants reported it would be most useful/helpful to see PACE labelling placed on sugary/fizzy drinks (79%), energy drinks (66%), milkshakes/frappes (54%) and alcoholic drinks (52%). S2 Table in S1 File. Cross tabulation of multiple responses identified that fizzy drinks, energy drinks, chocolate bars and cakes/pastries bought in fast food outlets, supermarkets and takeaway locations were the most popular combinations of food/drinks by locations that participants would like to see PACE labelling displayed. S3 Table in S1 File.

### Potential negative effects of PACE labelling and predictor variables

Of all participants (n = 2,668), 50% (n = 1,237) thought that seeing PACE labels on food/drinks would definitely/probably make them think they needed to ‘burn off’ all the calories they consumed each day, with 48% (n = 1,381) responding that this would not really or definitely would not be the case for them. Forty percent (n = 967) thought that definitely/probably seeing PACE labels on food/drinks would make them feel anxious about their weight, 58% (n = 1,658) responded not really/definitely not and 2% (n = 43) were unsure. Of participants who perceived they were underweight (n = 126), 33% (n = 41) responded definitely/probably and 67% (n = 85) not really/definitely not (weighted results). Age, gender and weight perception were significantly associated with agreement that PACE labels would result in feelings of anxiety about weight. This was more likely in younger age groups, females and being perceived overweight. Being in the 18–34 years age group was the most predictive factor (weighted adjusted OR = 2.69 (1.67 to 4.35)). Table 2.

## Discussion

Data from this UK nationally representative survey found most participants thought that traffic light food labels were easy to understand, but~40% reported they never/rarely stopped them buying/eating food/drinks high in calories and most did not look at them regularly. PACE labelling was considered easy to understand and most participants thought that if PACE labels were placed on food/drinks they would be likely to look at them to help them decide what to buy/eat. In direct head-to-head comparisons more participants preferred traffic light labelling, although PACE was considered easier to understand, more likely to catch their attention and to stop them buying food/drinks that were high in calories. There was a preference for PACE labelling to be placed on discretionary foods, typically (i.e., chocolate & cakes), rather than ‘every day’ food items (i.e., bread, pasta, fruit & vegetables), and a preference for PACE to be displayed in fast food outlets, supermarkets, takeaway/online menus and vending machines, all locations that typically sell high energy dense food/drinks. Shifts in food consumption patterns and relatively low nutritional literacy amongst the public may provide justification for considering the introduction of alternative labelling approaches such as PACE.

### Food labelling

Consistent with previous studies we found that a large proportion of the public do not look at nutrition labelling, in this case traffic light labelling, because they find such labelling difficult to understand [9, 18] This is concerning because in many developed countries most of the population are living with being overweight [19], and it is therefore critical that nutritional information is expressed to the public in a format that allows them to make an informed decision about what they consume. Of note, women and physically active participants (five or more days/week) were more likely to use traffic light labelling to prevent them for purchasing high calorie food/drinks. This result is in line with other research demonstrating that women are more health conscious than men [20]. It was interesting to note participants who were least physically active were less likely to use traffic light labelling. It may be that if PACE labelling were to be introduced, it would serve as a nudge to encourage these individuals to engage in physical activity more regularly.

### Direct comparisons of traffic light and PACE labelling

Whilst more participants preferred traffic light to PACE labelling (10% more), there was also support for PACE as a new approach to labelling food. Specifically, participants reported PACE was easier for them understand and more likely to prevent them from buying high calorie food/drinks than traffic light labelling, which may in turn be more helpful in guiding the public towards healthier food selections. Several socio-economic demographic factors increased the likelihood that PACE labels would be considered useful in helping people decide what to buy/eat, but the perception of being overweight was the most predictive. This highlights that people who are living with being overweight may find additional information in the form of PACE useful in helping them select the right types of foods to help them manage their weight. Our findings that the public are open to the implementation of interpretational food labelling, such as PACE labelling, is consistent with previous research [21], but consideration should be given to other research involving smaller samples which found the public prefer calorie over PACE information [22] and other studies that have indicated PACE may not be effective [23]. Furthermore, PACE labelling may be less informative overall because detailed nutritional information is typically not displayed (sugars, fats, & protein etc) on this type of labelling. As such, PACE labelling may be useful in facilitating weight management and/or promoting physical activity, but it may be less useful in encouraging healthy food choices, which involves information about several nutrition components. Of note here, in the free text comments, there was a strong preference for a combination of both traffic light and PACE labelling to be used and this combined approach would address the concern that PACE labelling does not including additional nutritional information. This combined approach would not be onerous to include within food labelling systems either.

### Locations and types of food for PACE labelling

PACE labelling was thought to be most helpful/useful displayed in fast food restaurants, supermarkets, on takeaway/online menus and vending machines. Except for supermarkets, these are locations (particularly fast food) that are associated with high energy dense items. Ready-to-eat food prepared away from the home and prepared foods are typically higher in saturated fat and calories, of poor nutritional quality and served in larger proportions than home prepared food and therefore a key contributing factor to over consumption and diet related diseases [24, 25]. Together, this data highlights the strong need for persuasive food labelling to be placed in out-of-home food/eating environments, and it is these contexts that PACE labelling might be most beneficial. The use of digital menu boards is becoming more common place in out of home eating settings, particularly given the increase in calorie labelling legislation in developed world countries. PACE labelling could therefore be relatively easy to implement without incurring substantial costs, and the food industry might want to engage with this strategy to show that they can be part of the solution to reduce obesity rates. Participants thought PACE labelling would be most helpful to them on chocolate bars/cakes/pastries, ready meals, sweets, crisps, biscuits, sugary/fizzy drinks, energy drinks, milkshakes/frappes and alcohol, all discretionary energy dense items that are often consumed outside the home setting as snacks, rather than food that is more nutritious. Moreover, we know that even small reductions in discretionary food consumption is likely to have substantial health benefits, and PACE labelling may be able to facilitate these nudge changes [26].

### Negative effects of PACE labelling

Concerns have been raised about the possibility that PACE labelling may contribute to disordered eating [27]. Most participants did not believe PACE labelling would make them feel anxious about their weight, although a significant proportion (40%) were concerned. The 18–34 year age group, being female and perceived to be overweight were the strongest predictive factors of believing PACE labelling would lead to anxious thoughts about weight, highlighting that some population groups may need additional attention were PACE labelling implemented.

This study was also interested in participants views about whether PACE labelling would negatively impact their views about physical activity. A preliminary study that recruited a small sample (n = 469) found those exposed to PACE labelling as part of a hypothetical experimental study perceived exercise as less enjoyable than participants not exposed [28]. It may be that PACE could increase motivation to participate in physical activity and provide a visual ‘shock’ about the high amount of physical activity often required in food selections, leading to the selection of lower calorie items. This is turn could in turn impact the supply and demand chain leading to food companies offering more lower calorie alternatives to consumers. However, it may also the case that PACE could reduce motivation for physical activity, leading to individuals feeling less positive and perceiving physical activity as futile given the large amount of physical activity required to expend the calories in food consumed. Some consideration also should be given to the possibility that PACE labelling results in reinforcing that view that physical activity is an unpleasant experience that is completed for the purpose of expending calories. It is also possible that PACE leads to people using the selection of lower calorie food as a rationale for not being physically active each day, compromising their health. In this study, the evidence was divided about whether PACE labels would be likely to make them feel they needed to ‘burn off’ all the calories they consumed each day. Together, these findings suggest that if PACE labelling were to be introduced into food settings, it would be important to highlight to the public its specific purpose, whilst also emphasising the broader importance of physical activity for optimal health.

### Strengths and limitations

To our knowledge, this is the first study to assess the views of consumers about PACE labelling in a nationally representative sample. Previous studies have tended to focus on sub-populations in specific geographical areas. The use of a large national panel and a high response rate should reduce potential concerns for response bias, and survey weights were applied in analyses to account for any response differences. It is possible that respondents to an online survey may differ from the general population, although 92% of UK adults used the internet in 2021 [29]. Nevertheless, a particular strength of this study is the use of a Knowledge Panel, where panellists who do not have an easy way to access to the internet (often those on the lowest incomes), are given resources to ensure that a lack of access is not a barrier to such individuals being able to express their views in studies such as this one. Participants were given opportunities to comment further on their survey responses and most took the opportunity to do so. In total we obtained 1,095 open text comments to the reasons why participants preferred a particular label, and a further 821 general comments about food labelling, providing us with a substantial amount of rich contextual descriptive data, to supplement the quantitative survey findings.

Despite several strengths, the current study has several important limitations. There may be important differences in attitudes and views between responders and non-responders, although our survey had a relatively high response rate at 67%. As PACE labelling is not routinely implemented in the United Kingdom, or indeed any other countries, we were asking participants to comment on hypothetical PACE labelling scenarios. Participants responded to pictures of food labels within an online survey, and we may have recorded different responses had we presented them with real food items that showed different types of labelling on the packaging/menus. We conducted an observational study which only allow us to present associations between data.

### Implications

This study makes several important contributions to the evidence regarding the views of the public about traffic light labelling and the acceptability and potential implementation PACE labelling in food settings. Most participants reported that traffic light labelling was easy to understand, but many reported this type of labelling was not effective is preventing them from buying/eating food/drinks that were high in calories, and most did not look at them regularly. These results highlight a need for consideration to be given to the implementation of strategies that encourage the public to be more engaged with food labelling. PACE labelling was considered easy to understand and most participants thought that if it were implemented, they would be likely to use it to help them decide what to buy/eat. As PACE labelling includes both calorie and physical activity equivalent information, our findings may inform recent legislative changes in many countries mandating that calorie labelling is displayed in cafes, restaurants and takeaways, non-prepacked food/soft drinks and out of home settings. There was a high level of acceptability for PACE labelling to be displayed in fast food outlets, supermarkets, takeaway/online menus and vending machines, typically locations that sell high energy dense food/drinks, and which contribution substantially to overweight and obesity in the population. This study highlights there is a low level of concern amongst the public that PACE labelling will lead to the development an unhealthy relationship with food. The implementation of PACE labelling, as an example of interpretive nutritional labelling, may act as a nudge for the food industry to resize and/or reformulate their food products, particularly those high in calories.

### Conclusion

Overweight is a serious public health concern in many countries and the World Health Organisation views nutrition labelling as a critical part of its strategy on diet, physical activity and health. A key public health ambition is to increase the availability of nutrition information on labels for foods eaten and prepared away from home, and in this study there was a preference for PACE labelling to be placed on discretionary foods. The public indicated that they saw value to their health in labelling food with PACE information which may support its implementation as a strategy to prevent and reduce overweight in the population. Our findings show that PACE labelling may be a potentially important health policy-based approach to strengthen and complement current approaches to food labelling.

## Supporting information

S1 File (DOCX)

## References

[1] World Health Organisation (2020) Obesity. https://www.who.int/news-room/facts-in-pictures/detail/6-facts-on-obesity#:~:text=Obesity%20has%20reached%20epidemic%20proportions,of%20being%20overweight%20or%20obese (accessed 14^th^ October 2020).

[2] Swinburn B, Egger G, Raza F (1999) Dissecting obesogenic environments: the development and application of a framework for identifying and prioritizing environmental interventions for obesity. *Prev Med* 29, 563–570. doi: 10.1006/pmed.1999.0585 10600438

[3] Food Standards Agency (2018) Nutrition labelling. https://www.food.gov.uk/business-guidance/nutrition-labelling#front-of-pack-nutritional-labelling (accessed 24^th^ December 2021).

[4] World Health Organization (2002) Global strategy on diet, physical activity, and health. http://www.who.int/dietphysicalactivity/strategy/eb11344/strategy_english_web.pdf (accessed 27^th^ November 2018).

[5] Zlatevska N, Neumann N, Dubelaar C (2018) Mandatory calorie disclosure: A comprehensive analysis of its effect on consumers and retailers. *J Retailing* 94, 89–101. 10.1016/j.jretai.2017.09.007

[6] Crockett RA, King SE, Marteau TM et al. (2018) Nutritional labelling for healthier food or non-alcoholic drink purchasing and consumption. *Cochrane Database Syst Rev* 2, CD009315. doi: 10.1002/14651858.CD009315.pub2 29482264 PMC5846184

[7] Bleich SN, Economos CD, Spiker M et al. (2017) A systematic review of calorie labeling and modified calorie labeling interventions: Impact on consumer and restaurant behavior. *Obesity* 25, 2018–2044. doi: 10.1002/oby.21940 29045080 PMC5752125

[8] Long MW, Tobias DK, Cradock AL et al. (2015) Systematic review and meta-analysis of the impact of restaurant menu calorie labeling. *Am J Public Health* 105, e11–24. doi: 10.2105/AJPH.2015.302570 25790388 PMC4386504

[9] Krukowski RA, Harvey-Berino J, Kolodinsky J et al. (2006) Consumers may not use or understand calorie labeling in restaurants. *J Am Diet Assoc* 106, 917–920. doi: 10.1016/j.jada.2006.03.005 16720133

[10] Block JP, Condon SK, Kleinman K et al. (2013) Consumers’ estimation of calorie content at fast food restaurants: cross sectional observational study. *BMJ* 346, f2907. doi: 10.1136/bmj.f2907 23704170 PMC3662831

[11] Daley AJ, McGee E, Bayliss S et al. (2020). Effects of physical activity calorie equivalent food labelling to reduce food selection and consumption: systematic review and meta-analysis of randomised controlled studies. *J Epidemiol Community Health* 74, 269–275. doi: 10.1136/jech-2019-213216 31822568

[12] Thaler R., & Sunstein C. (2008). *Nudge*: *Improving decisions about health*, *wealth*, *and happiness*. New Haven: Yale University Press.

[13] Department of Health and Social Care (2021). Calorie labelling in the out of home sector: implementation guidance. https://www.gov.uk/government/publications/calorie-labelling-in-the-out-of-home-sector/calorie-labelling-in-the-out-of-home-sector-implementation-guidance (accessed 2^nd^ January 2022).

[14] Rosenbaum S (2011) The Patient Protection and Affordable Care Act: implications for public health policy and practice. *Public Health Rep* 126, 130–135. doi: 10.1177/003335491112600118 21337939 PMC3001814

[15] Mori Ipsos (2021) UK KnowledgePanel. https://www.ipsos.com/en-uk/uk-knowledgepanel# (accessed 4^th^ March 2021).

[16] McLennan D, Barnes H, Noble M et al. (2011) The English Indices of Deprivation 2010. Department for Communitites and Local Government. https://www.gov.uk/government/uploads/system/uploads/attachment_data/file/6320/1870718.pdf (accessed 23^rd^ April 2019).

[17] Office for National Statistics (2021) Population estimates. https://www.ons.gov.uk/peoplepopulationandcommunity/populationandmigration/populationestimates (accessed 27^th^ April 2022).

[18] Mhurchu CN & Gorton D (2007) Nutrition labels and claims in New Zealand and Australia: a review of use and understanding. *Aust N Z J Public Health* 31, 105–112. doi: 10.1111/j.1753-6405.2007.00026.x 17460999

[19] World Health Organization (2020) Obesity and overweight. https://www.who.int/news-room/fact-sheets/detail/obesity-andoverweight (accessed 26^th^ March 2021).

[20] Glanz K, Basil M, Maibach E et al. (1998) Why Americans eat what they do: taste, nutrition, cost, convenience, and weight control concerns as influences on food consumption. *J Am Diet Assoc* 98, 1118–1126. doi: 10.1016/S0002-8223(98)00260-0 9787717

[21] Bleich SN & Pollack KM (2010) The publics’ understanding of daily caloric recommendations and their perceptions of calorie posting in chain restaurants. *BMC Public Health* 10, 121. doi: 10.1186/1471-2458-10-121 20214811 PMC2847976

[22] Fitch RC, Harnack LJ, Neumark-Sztainer DR et al. (2009) Providing calorie information on fast-food restaurant menu boards: consumer views. *Am J Health Promot* 24, 129–132. doi: 10.4278/ajhp.08031426 19928485 PMC2964072

[23] Reynolds JP, Ventsel M, Kosīte D, Dames BR, Brocklebank L, Masterton S, et al. 2021. Impact of decreasing the proportion of higher energy foods and reducing portion sizes on food purchased in worksite cafeterias: A stepped-wedge randomised controlled trial. *PLOS Med* 18(9), e1003743. Available from: https://journals.plos.org/plosmedicine/article?id=10.1371/journal.pmed.1003743 34520468 10.1371/journal.pmed.1003743 PMC8439477

[24] Variyam J. Nutrition labeling in the food-away-from-home sector: An economic assessment. United States Department of Agriculture. Economic Research Report No. 4; 2005.

[25] Lachat C, Nago E, Verstraeten R, et al. (2012). Eating out of home and its association with dietary intake: a systematic review of the evidence. *Obes Rev*, 13, 329–346. doi: 10.1111/j.1467-789X.2011.00953.x 22106948

[26] Lal A, Peeters A, Brown V et al. (2020) The Modelled Population Obesity-Related Health Benefits of Reducing Consumption of Discretionary Foods in Australia. *Nutrients* 12, 649. doi: 10.3390/nu12030649 32121199 PMC7146305

[27] McGeown L (2019) The calorie counter-intuitive effect of restaurant menu calorie labelling. *Can J Public Health* 110, 816–820. doi: 10.17269/s41997-019-00183-7 30701412 PMC6964477

[28] Lee MS & Thompson JK (2016) Exploring enhanced menu labels’ influence on fast food selections and exercise-related attitudes, perceptions, and intentions. *Appetite* 105, 416–422. doi: 10.1016/j.appet.2016.06.007 27289007

[29] Office for National Statistics (2021) Internet users, UK: 2020 https://www.ons.gov.uk/businessindustryandtrade/itandinternetindustry/bulletins/internetusers/2020 (accessed 27th April 2022).

