# Supplementary material for: Implementing physical activity calorie equivalent (PACE) food labelling: Views of a nationally representative sample of adults in the United Kingdom
Source: PLoS One. 2023 Sep 14;18(9):e0290509. doi: 10.1371/journal.pone.0290509 (PMC10501579; doi:10.1371/journal.pone.0290509)
Supplement: S1 File — (DOCX) [file pone.0290509.s001.docx]

**Implementing physical activity calorie equivalent (PACE) food labelling: Views of a nationally representative sample of adults in the United Kingdom**

Amanda J Daley^1^ Professor of Behavioural Medicine [https://orcid.org/0000-0002-4866-8726](about:blank)

Victoria E Kettle^1^ Research Associate [https://orcid.org/0000-0002-6676-3444](about:blank)

Andrea K Roalfe^1^ Medical Statistician [https://orcid.org/0000-0003-1622-2639](about:blank)

^1^ Centre for Lifestyle Medicine and Behaviour (CLiMB), The School of Sport, Exercise and Health Sciences, Loughborough University, UK

**Corresponding author**

Professor Amanda Daley

Centre for Lifestyle Medicine and Behaviour (CLiMB)

The School of Sport, Exercise and Health Sciences

Loughborough University,

Email: a.daley@lboro.ac.uk

**Table S1. Weighted multinomial regression models for comparison of views**

| Preference category | Demographic Characteristic | Labelling | Easier to understand | Catches attention | Avoid high calorie food and drinks |
| --- | --- | --- | --- | --- | --- |
|  |  | Weighted Adjusted Relative Risk Ratio  (95% CI) | Weighted Adjusted Relative Risk Ratio  (95% CI) | Weighted Adjusted Relative Risk Ratio  (95% CI) | Weighted Adjusted Relative Risk Ratio  (95% CI) |
| Traffic light labelling (base outcome) |  |  |  |  |  |
| PACE labelling |  |  |  |  |  |
|  | Age group |  |  |  |  |
|  | 18-34 (reference) | 1.00 | 1.00 | 1.00 | 1.00 |
|  | 35-44 | 1.27 ( 0.85 to 1.89) | 1.03 (0.66 to 1.58) | 0.96 (0.65 to 1.44) | 0.75 (0.50 to 1.14) |
|  | 45-54 | **1.49 (1.02 to 2.16)** | 1.14 (0.76 to 1.71) | 1.01 (0.70 to 1.45) | 0.93 (0.63 to 1.38) |
|  | 55-64 | 1.31 (0.92 to 1.87) | 1.09 (0.74 to 1.61) | 1.12 (0.79 to 1.57) | 0.85 (0.59 to 1.24) |
|  | 65+ | 0.91 (0.64 to 1.30) | 0.83 (0.57 to 1.22) | 0.88 (0.63 to 1.24) | **0.60 (0.42 to 0.87)** |
|  |  |  |  |  |  |
|  | Gender |  |  |  |  |
|  | Male (reference) | 1.00 | 1.00 | 1.00 | 1.00 |
|  | Female | 0.94 (0.75 to 1.19) | 1.02 (0.80 to 1.30) | 0.92 (0.74 to 1.15) | 1.04 (0.82 to 1.31) |
|  |  |  |  |  |  |
|  | Perception of weight |  |  |  |  |
|  | Underweight (reference) | 1.00 | 1.00 | 1.00 | 1.00 |
|  | About right | 0.83 (0.42 to 1.66) | 1.09 (0.52 to 2.27) | 1.58 (0.82 to 3.05) | 1.46 (0.68 to 3.13) |
|  | overweight | 1.10 (0.56 to 2.17) | 1.26 (0.61 to 2.59) | 1.49 (0.78 to 2.85) | 1.72 (0.83 to 3.63) |
|  |  |  |  |  |  |
|  | Physical activity (days/week) |  |  |  |  |
|  | 0 (reference) | 1.00 | 1.00 | 1.00 | 1.00 |
|  | 1-2 | 0.90 (0.63 to 1.28) | 0.97 (0.67 to 1.41) | 1.05 (0.74 to 1.48) | 0.92 (0.64 to 1.32) |
|  | 3-4 | 1.00 (0.70 to 1.43) | 1.05 (0.72 to 1.54) | **1.42 (1.00 to 2.00)** | 1.20 (0.83 to 1.73) |
|  | 5+ | 1.25 (0.88 to 1.77) | 0.99 (0.68 to 1.44) | **1.45 (1.03 to 2.05)** | 1.13 (0.78 to 1.64) |
|  |  |  |  |  |  |
|  | constant | 0.65 (0.30 to 1.37) | 1.25 (0.53 to 2.96) | 0.90 (0.42 to 1.02) | 1.08 (0.45 to 2.58) |
| About the same |  |  |  |  |  |
|  | Age group |  |  |  |  |
|  | 18-34 (reference) | 1.00 | 1.00 | 1.00 | 1.00 |
|  | 35-44 | 0.95 (0.58 to 1.56) | 0.77 (0.48 to 1.22) | 0.79 (0.46 to 1.36) | 0.58 (0.34 to 1.02) |
|  | 45-54 | 0.92 (0.57 to 1.48) | 0.85 (0.56 to 1.31) | 0.74 (0.44 to 1.24) | 0.93 (0.56 to 1.55) |
|  | 55-64 | 1.10 (0.70 to 1.72) | 0.92 (0.62 to 1.38) | 0.80 (0.49 to 1.28) | 0.92 (0.57 to 1.48) |
|  | 65+ | 1.07 (0.69 to 1.65) | **0.67 (0.45 to 1.00)** | 1.00 (0.63 to 1.59) | 0.65 (0.41 to 1.04) |
|  |  |  |  |  |  |
|  | Gender |  |  |  |  |
|  | Male (reference) | 1.00 | 1.00 | 1.00 | 1.00 |
|  | Female | 0.94 (0.70 to 1.25) | 1.24 (0.95 to 1.61) | 1.01 (0.73 to 1.40) | 1.06 (0.78 to 1.45) |
|  |  |  |  |  |  |
|  | Perception of weight |  |  |  |  |
|  | Underweight (reference) | 1.00 | 1.00 | 1.00 | 1.00 |
|  | About right | 1.80 (0.56 to 5.76) | **2.78 (1.29 to 6.00)** | 1.82 (0.57 to 5.78) | **2.93 (1.21 to 7.11)** |
|  | overweight | 2.08 (0.66 to 6.53) | **2.45 (1.15 to 5.24)** | 1.27 (0.41 to 3.91) | **2.76 (1.15 to 6.62)** |
|  |  |  |  |  |  |
|  | Physical activity (days/week) |  |  |  |  |
|  | 0 (reference) | 1.00 | 1.00 | 1.00 | 1.00 |
|  | 1-2 | 1.26 (0.81 to 1.94) | 0.77 (0.52 to 1.16) | 0.85 (0.54 to 1.36) | 1.07 (0.67 to 1.72) |
|  | 3-4 | 1.26 (0.80 to 1.98) | 1.39 (0.93 to 2.09) | 1.02 (0.61 to 1.68) | 1.43 (0.90 to 2.27) |
|  | 5+ | 1.33 (0.86 to 2.07) | 0.91 (0.60 to 1.36) | 1.01 (0.63 to 1.61) | 1.05 (0.65 to 1.69) |
|  |  |  |  |  |  |
|  | constant | **0.16 (0.05 to 0.55)** | 0.45 (0.19 to 1.10) | 0.37 (0.11 to 1.20) | **0.20 (0.07 to 0.54)** |
| Neither |  |  |  |  |  |
|  | Age group |  |  |  |  |
|  | 18-34 (reference) | 1.00 | 1.00 | 1.00 | 1.00 |
|  | 35-44 | 0.52 (0.20 to 1.39) | 0.83 (0.23 to 2.93) | 2.13 (0.68 to 6.67) | 0.69 (0.34 to 1.39) |
|  | 45-54 | 0.90 (0.45 to 1.80) | 1.40 (0.47 to 4.20) | 1.94 (0.67 to 5.59) | 1.08 (0.58 to 2.03) |
|  | 55-64 | 0.95 (0.49 to 1.83) | 1.29 (0.44 to 3.77) | **3.70 (1.41 to 9.70)** | 1.48 (0.83 to 2.66) |
|  | 65+ | 1.50 (0.81 to 2.77) | 2.45 (0.95 to 6.35) | **5.01 (1.98 to 12.72)** | **1.94 (1.10 to 3.40)** |
|  |  |  |  |  |  |
|  | Gender |  |  |  |  |
|  | Male (reference) | 1.00 | 1.00 | 1.00 | 1.00 |
|  | Female | **0.41 (0.27 to 0.64)** | **0.38 (0.21 to 0.69)** | **0.37 (0.23 to 0.59)** | **0.53 (0.38 to 0.75)** |
|  |  |  |  |  |  |
|  | Perception of weight |  |  |  |  |
|  | Underweight (reference) | 1.00 | 1.00 | 1.00 | 1.00 |
|  | About right | 2.18 (0.80 to 5.89) | 1.68 (0.50 to 5.72) | 1.06 (0.40 to 2.76) | 0.94 (0.39 to 2.23) |
|  | overweight | 1.69 (0.65 to 4.41) | 0.93 (0.27 to 3.14) | 0.93 (0.35 to 2.46) | 0.60 (0.26 to 1.40) |
|  |  |  |  |  |  |
|  | Physical activity (days/week) |  |  |  |  |
|  | 0 (reference) | 1.00 | 1.00 | 1.00 | 1.00 |
|  | 1-2 | **0.47 (0.26 to 0.85)** | 0.51 (0.22 to 1.19) | 0.59 (0.32 to 1.09) | **0.55 (0.34 to 0.90)** |
|  | 3-4 | 0.80 (0.43 to 1.50) | **0.39 (0.17 to 0.90)** | 0.76 (0.39 to 1.49) | 0.74 (0.43 to 1.25) |
|  | 5+ | 0.99 (0.57 to 1.72) | 0.83 (0.41 to 1.66) | 0.78 (0.44 to 1.38) | 0.73 (0.45 to 1.17) |
|  |  |  |  |  |  |
|  | constant | **0.15 (0.06 to 0.40)** | **0.13 (0.03 to 0.51)** | **0.11 (0.03 to 0.38)** | 0.74 (0.26 to 2.14) |

Statistical significant effects (p<0.05) are in bold.

Constant: baseline relative risk for each outcome; CI: confidence interval.

**Table S2: Preferred types of food/drinks for PACE labelling and locations for PACE to be displayed**

| **Food** | **%** | **Drinks** | **%** | **Locations** | **%** |
| --- | --- | --- | --- | --- | --- |
| Chocolate bars | 60 | Sugary/fizzy drinks | 79 | Fast food | 68 |
| Cakes/pastries | 57 | Energy drinks | 66 | Supermarkets | 67 |
| Ready meals | 54 | Milkshakes/frappes | 54 | Takeaway/online menus | 55 |
| Sweets | 46 | Alcoholic drinks | 52 | Vending machines | 39 |
| Crisps | 41 | Smoothies | 38 | Restaurant menus | 35 |
| Biscuits | 41 | Fruit juices | 28 | Coffee shops | 29 |
| Prepared sandwiches | 28 | Speciality coffees | 26 | Petrol stations | 21 |
| Pizza/garlic bread | 27 | Hot chocolate | 21 | Pubs | 19 |
| Pies | 22 | Cordial | 11 | Canteens | 16 |
| Cereal | 8 | Milk | 6 |  |  |
| Cheese | 7 | English tea/coffee | 2 |  |  |
| Bread, pasta, potatoes | 3-5 | Herbal teas/water | 1 |  |  |
| Fruit, vegetables, meat | 2-3 |  |  |  |  |

Five response option were permitted. % are weighted values

**Table S3: Most popular location and food/drink combinations for PACE labelling to be displayed**

| **Location** | **Food/drink** | **Frequency (%)** | **Weighted %** |
| --- | --- | --- | --- |
| Fast food | Sugary fizzy drinks | 1659 (72.1%) | 70.2% |
| Supermarkets | Sugary fizzy drinks | 1580 (68.7%) | 69.3% |
| Fast food | Energy drinks | 1411 (61.3%) | 58.8% |
| Takeaway/online menu | Sugary fizzy drinks | 1375 (59.8%) | 56.1% |
| Supermarkets | Energy drinks | 1329 (57.8%) | 58.6% |
| Fast food | Chocolate bars | 1204 (53.1%) | 54.0% |
| Fast food | Cakes/pastries | 1200 (52.9%) | 51.7% |
| Fast food | Ready meals | 1198 (52.8%) | 49.8% |
| Supermarkets | Chocolate bars | 1183 (52.2%) | 54.3% |
| Supermarkets | Cakes/pastries | 1166 (51.4%) | 51.4% |
| Supermarkets | Ready meals | 1145 (50.5%) | 49.8% |

Includes only those who responded (n=2,292 location by food; n=2,301 location by drink). Five response were permitted. N=actual numbers of responses and % are weighted values

**Supplementary Material S1**

Open text from participants who preferred traffic light labelling: thematic analysis

Of participants who preferred traffic light labelling and provided an open text comment (n=1,122), the main reasons given were traffic light labelling contains more information/detail (n=651), clear/simple/easier to understand (n=210), bold bright colours give immediate information about food (n=187), more accustomed to traffic light labelling (n=72), prefer traffic light because PACE could be misunderstood/trigger negative health consequences (eating disorders/over exercise make people feel guilty/anxious) (n=69) and traffic light was more relevant for medical conditions/needs/particular diets/allergies (n=60).

Open text from participants who preferred PACE labelling: thematic analysis

For participants who preferred PACE labelling and provided an open comment, (n=783), the most common reasons (theme) given were; PACE was clear/simple/easy to understand (n=482), it provided a context and implications for food and was therefore more meaningful (n=128), PACE would encourage more physical activity (n=109), was more eye catching, bolder/brighter and easier to see (n=103), at a glance PACE was more likely to inform consumers what they needed to know (n=50), PACE would be more likely to make people consider more carefully what they were eating/purchasing (n=44) and PACE labelling highlighted the importance of physical activity (n=18).

General open text comments

All participants were offered the opportunity to make any additional open text comments they wished to make about food labelling and 821 were received and thematically collated as follows; PACE and traffic light labelling should be combined (n=127), PACE labelling alone lacks nutritional information (n=66), PACE is clear and easy to understand (n=30), PACE labelling may lead to disordered eating and the possibility people may over exercise to compensate for their calorie consumption each day (n=48), PACE was a good idea (n=44) and healthy eating is more than simply about calories (n=25).
